# Two cryptic species of California mustard within *Caulanthus lasiophyllus*

**DOI:** 10.1002/ajb2.1562

**Published:** 2020-12-28

**Authors:** Justen B. Whittall, Timothy M. Butler, Cynthia Dick, Brody Sandel

**Affiliations:** ^1^ Department of Biology Santa Clara University 500 El Camino Real Santa Clara California 95053 USA

**Keywords:** annual precipitation, Brassicaceae, California mustard, chloroplast *ndh*F gene, chloroplast *trn*L‐F region, internal transcribed spacer region, mean temperature, morphological analysis

## Abstract

**Premise:**

Cryptic species are evolutionarily distinct lineages lacking distinguishing morphological traits. Hidden diversity may be lurking in widespread species whose distributions cross phylogeographic barriers. This study investigates molecular and morphological variation in the widely distributed *Caulanthus lasiophyllus* (Brassicaceae) in comparison to its closest relatives.

**Methods:**

Fifty‐two individuals of *C. lasiophyllus* from across the species’ range were sequenced for the nuclear ribosomal internal transcribed spacer region (ITS) and the chloroplast *trn*L‐F region. A subset of these samples were examined for the chloroplast *ndh*F gene. All 52 individuals were scored for 13 morphological traits, as well as monthly and annual climate conditions at the collection locality. Morphological and molecular results are compared with the closest relatives*—C. anceps* and *C. flavescens*—in the “Guillenia Clade.” To test for polyploidy, genome size estimates were made for four populations.

**Results:**

*Caulanthus lasiophyllus* consists of two distinct lineages separated by eight ITS differences—eight times more variation than what distinguishes *C. anceps* and *C. flavescens*. Fewer variable sites were detected in *trn*L‐F and *ndh*F regions, yet these data are consistent with the ITS results. The two lineages of *C. lasiophyllus* are geographically and climatically distinct; yet morphologically overlapping. Their genome sizes are not consistently different.

**Conclusions:**

Two cryptic species within *C. lasiophyllus* are distinguished at the molecular, geographic, and climatic scales. They have similar genome sizes and are morphologically broadly overlapping, but an ephemeral basal leaf character may help distinguish the species.

Cryptic species are evolutionarily distinct lineages with few or no distinguishable morphological traits (Clausen, 1951; Grant, 1981; Paris et al., 1989; Whittall et al., 2004). They are predicted to be concentrated in hyperdiverse families where subtle morphological or ecological differences often reflect strong reproductive isolation (Chan et al., 2001; Baldwin, 2006; Yost et al., 2012; Johnson et al., 2013). Within these families, cryptic diversity is most likely lurking in widespread species whose distributions span phylogeographic barriers to gene flow (Shaw, 2000; Baunsteiger et al., 2012; Highton, 2014). Cryptic species are most commonly diagnosed by their exceptional levels of molecular distinctiveness in light of their relative lack of morphological differentiation (Baldwin, 2006; Rowsey and Egge, 2017).

A hidden layer of cryptic biodiversity is emerging in animal and plant studies in the California Floristic Province (Carlsbeek et al., 2003; Angert and Schemske, 2005; Baldwin, 2006; LaPointe and Rissler, 2006; Crummett and Eernisse, 2007; Graves and Schrader, 2008; Carter, 2012; Johnson et al., 2013; Highton, 2014; Reilly and Wake, 2015; Emata and Hedin, 2016). One of 25 global biodiversity hotspots (Myers et al., 2000), the California Floristic Province harbors approximately 6100 native vascular plant taxa (Baldwin et al., 2012) of which greater than 40% are endemic (Burge et al., 2016). This diversity is driven by California’s unique physiography producing steep ecological gradients (Stebbins and Major, 1965; Anacker and Harrison, 2012; Lancaster and Kay, 2013; Harrison, 2013) and numerous phylogeographic patterns (Carlsbeek et al., 2003; Baldwin, 2014; Thornhill et al., 2017). Together, these factors increase diversification through increased rates of speciation and decreased rates of extinction (Anacker et al., 2011; Lancaster and Kay, 2013).

The Brassicaceae is one of the six largest families in the California Floristic Province (Baldwin et al., 2012). Although recent molecular phylogenetic analyses of the family have clarified tribal and some generic relationships (Beilstein et al., 2010; Guo et al., 2017), many lineages are still poorly understood and often contain species that are only differentiated by subtle morphological characters such as trichome type, leaf shape, and fruit morphology (Beilstein et al., 2006; Baldwin et al., 2012). The Thelypodieae harbors several diverse and taxonomically challenging genera such as *Streptanthus* Nutt. and *Caulanthus* S. Watson (Buck, 1995; Al‐Shehbaz et al., 2006; Warwick et al., 2010; Al‐Shehbaz, 2012; Cacho et al., 2014). Within *Caulanthus*, phylogenetic analyses consistently recover what has become known as the “Guillenia Clade” consisting of *C. lasiophyllus*, *C. anceps,* and *C. flavescens* (Cacho et al., 2014). It is sister to the ecologically and morphologically diverse *Streptanthus* Clade I (sensu Cacho et al., 2014; see Warwick et al., 2009 for *ndh*F support for two‐thirds of the “Guillenia Clade”). This lineage has been variously treated as *Guillenia* Greene (Hickman, 1993), *Streptanthus* (Al‐Shehbaz, 2012) and currently, *Caulanthus* (Al‐Shehbaz, 2010; Baldwin et al., 2012). Here, we use *Caulanthus* for consistency with the currently accepted taxonomy and refer to the “Guillenia Clade” in quotes to avoid any taxonomic confusion.

In search of cryptic diversity, we focus on one relatively neglected, widespread, and morphologically complex lineage of the “Guillenia Clade”—*C. lasiophyllus* (Hook. & Arn.) Payson. Of the three species in the “Guillenia Clade,” *C. lasiophyllus* is the most widely distributed and morphologically variable (Payson, 1922; Baldwin et al., 2012). It is distributed from British Columbia, east to southern Colorado and New Mexico and south to Baja California and Sonora, Mexico (Al‐Shehbaz, 2010). In California, it is present in 44 of the 58 counties (75%) (Baldwin et al., 2012). The taxonomic history illustrates the challenge of morphological variability across a broad geographic range. Briefly, the type specimen (as *Turritis lasiophylla*) was collected by David Douglas in Monterey, California (Hooker and Arnott, 1838). Greene treated it as *C. lasiophyllus* and described several morphologically unique and geographically distinct forms (Greene, 1891). In an attempt to capture these “broad groups which are somewhat localized geographically,” Payson described three varieties (Payson, 1922), which both Jepson (1936) and Munz (1959) acknowledge, but Jepson comments that they are “of little importance morphologically,” being defined primarily geographically (1936). Nearly every author who has treated *C. lasiophyllus* has called for further study based on its widespread distribution and complex morphological variation (Greene, 1891; Payson, 1922; Jepson, 1936; Al‐Shehbaz, 2010; Baldwin et al., 2012).

In comparison, the other two members of the “Guillenia Clade” are morphologically well‐defined California endemics (Munz, 1959; Baldwin et al., 2012). *Caulanthus anceps* is restricted to the southern end of the coast range (San Benito County south to Ventura County) and *C. flavescens* is found in the northern end of the coast range (San Benito County north to Glenn County). The latter is particularly common on serpentine soils (Baldwin et al., 2012). These two species have relatively large flowers (petals > 5 mm) and are likely facultative outcrossers compared to the reduced flowers of *C. lasiophyllus* (petals < 5 mm), which are likely autogamous (Baldwin et al., 2012).

Motivated by the morphological complexity of *C. lasiophyllus*, whose range includes broad climatic variation and several biogeographic barriers, we investigated the patterns of morphological, molecular, and climatic variation across the species’ range. Initially, there appeared to be two types based on their fruit orientation (erect vs. deflexed). This initial finding motivated a broader morphological and molecular survey across the species’ range in the field and in herbaria. Although fruit position was quickly identified as both variable within populations and developmentally plastic within individuals, the molecular phylogeographic investigation began to reveal two cryptic species within *C. lasiophyllus* (Whittall, 2011). These lineages are molecularly, geographically, and climatically distinct, yet broadly morphologically overlapping when compared to the two closest relatives in the “Guillenia Clade.”

## MATERIALS AND METHODS

### PCR and sequencing

**Figure 1:**
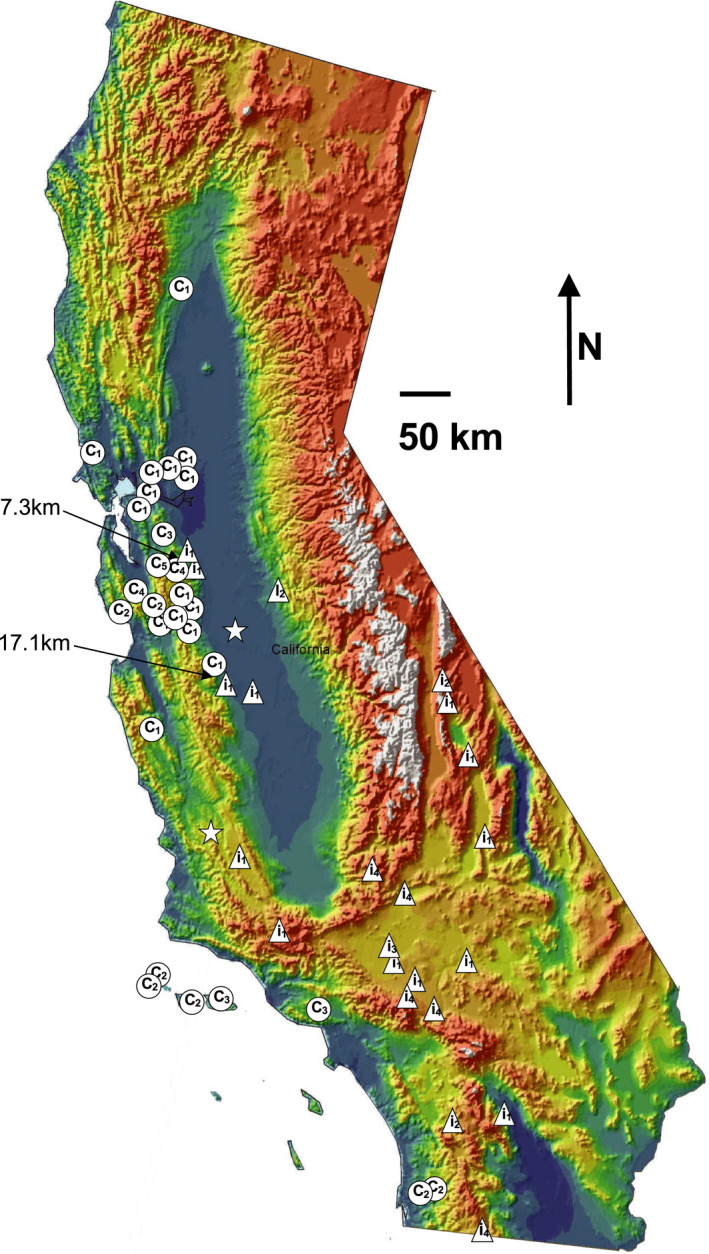
The location and type of *C. lasiophyllus* samples in California. Circles = northern/coastal type, triangles = southern/inland type, and stars = putative hybrids. Numbers inside symbols indicate the haplotype identifier (see Table 1). Four southern/inland samples outside of California are not shown (two from the inland regions of northern Baja California, Mexico—haplotype 4; one from southwestern Arizona—haplotype 5; one from southwestern Nevada—haplotype 4). Arrows point to the locations where the two types of *C. lasiophyllus* are geographically adjacent (distance between sampled populations indicated in kilometers).

We sampled 52 *C. lasiophyllus* individuals from across the species’ range in California representing half of the 44 counties where it has been recorded (Baldwin et al., 2012; Fig. 1, Appendix S1). Thirty‐seven of these originated from herbarium specimens (University and Jepson Herbaria, University of California [UC & JEPS], Berkeley, California, USA) and 15 samples were from fresh leaf tissue collected in the field (Collection Numbers JBW2007: 4210‐4224). We also included three samples of the closely related *C. anceps* and four samples of *C. flavescens*. We included ITS, *trn*L‐F and *ndh*F sequences of *Streptanthu*s *glandulosus* as an outgroup (Appendix S1). Genomic DNA was extracted from an average of 18 mg (6.5–43 mg) of dry leaf material from herbarium specimens as old as 1935 and approximately 100 mg of fresh leaf material collected in the field using the Qiagen DNeasy Plant Mini Kit (Carlsbad, California, USA). Tissue was homogenized at room temperature in 2 mL stainless steel tubes containing two ball bearings with the provided lysis buffer using a Mini‐Bead Beater (Biospec Products, Bartlesville, Oklahoma, USA).

#### ITS region

PCR amplification of the ITS region was conducted in 50 µL reaction volumes containing the following reagents (and their final concentrations): Fisher *Taq* Buffer B (1×), MgCl_2_ (2.5 mM), dNTPs (0.25 mM), ITS 5* forward primer (1 µM) (Liston et al., 1996), ITS 26S‐25R reverse primer (1 µM) (White et al., 1990), Fisher *Taq* polymerase (0.4 U) and 80 ng of DNA. Thermal cycling conditions began with a 94°C denature (2 min), followed by 35 cycles of 94°C (1 min), 50°C (45 s), and 72°C (45 s), ending with a final extension at 72°C (10 min). Polymerase chain reaction (PCR) products were then visualized on a 0.8% agarose gel stained with ethidium bromide. PCR products greater than or equal to 5 ng/µL were sent directly to sequencing on an ABI 3730 DNA sequencer (Sequetech, Santa Clara, California, USA) following the suggested BigDye protocol (Applied Biosystems, Foster City, California, USA). PCR products less than 5 ng/µL were gel purified (QIAquick gel extraction kit, Qiagen, Hilden, Germany), then used as template DNA for a second round of PCR amplification under the same conditions as indicated above. Each sample was directly sequenced in both the forward and reverse directions with the same primers as those used in PCR.

#### *trn*L‐F region

Each sample that was successfully sequenced for the ITS region was then amplified for the chloroplast *trn*L‐F region. Reaction conditions for the *trn*L‐F region were similar to those used to amplify the ITS region except for the primers used: *trn*C forward primer (1 µM) and *trn*F reverse primer (1 µM) (Taberlet et al., 1991). Thermal cycling conditions for the *trn*L‐F region were as follows: 94°C (2 min), 35 cycles of 94°C (10 s), 50°C (1 min), and 72°C (2 min), ending with a final extension of 72°C (5 min). Visualization and sequencing of the PCR products followed the same method as for the ITS region described above.

#### *ndh*F gene

A subset of 13 *C. lasiophyllus* samples based on the internal transcribed spacer (ITS) and *trn*L‐F results were amplified and sequenced for *ndh*F to increase the amount of chloroplast variable sites and further test the divergence found between the two types in the ITS region (Appendix S1). Two samples of *C. flavescens*, one sample of *C. anceps*, and one sample of *S. glandulosus* were also included. Reaction conditions for *ndh*F were similar to those used to amplify the ITS region except we amplified and sequenced this much larger gene using two pairs of primers (5F & 989R; 989F & 2100R; Beilstein et al., 2006). Each sample was sequenced with all four PCR primers.

### Sequence analysis

Forward and reverse reads were assembled into contigs using Sequencher (v. 4.8; Gene Codes Corporation, Ann Arbor, Michigan, USA) and all variable sites were validated in both forward and reverse chromatograms. The contigs were then exported to Bioedit (v. 7.0.9.0; Hall, 1999) for alignment. Sequences were aligned using the default settings in CLUSTALW (Thompson et al., 1994) ignoring gaps and then grouped by ITS and *trn*L‐F haplotype (identical sequences removed). Because sequence variation was low and homoplasy was rare, phylogenetic relationships among the unique haplotypes were analyzed using parsimony in PAUP (v. 4.0; Sinauer Associates, Sunderland, Massachusetts, USA). Branch support was determined from a bootstrap analysis with 1000 replicates. With substantially fewer samples for *ndh*F, it was included in a separate parsimony analysis after combining it with the ITS and *trn*L‐F regions for 15 samples with data from all three loci. Loci were aligned separately, then concatenated during the phylogenetic analysis in PAUP.

To estimate the geological setting when these two types of *C. lasiophyllus* diverged, we applied an approximate ITS molecular clock for annual plants (Kay et al., 2006). The mean number of pairwise differences between the northern‐coastal and southern‐inland haplotypes was used to determine the substitutions per site. Then, we applied an average ITS rate of 4.13 × 10^‒9^ substitutions per site per year (subs/site/yr) (Kay et al., 2006) to determine an approximate age of divergence.

### Genome sizing

To determine if polyploidy has played a role in the differentiation of the two types of *C. lasiophyllus*, plants with available seed representing both types were grown in the greenhouse and sampled for genome size estimation using flow cytometry (Benaroya Research Institute at Virginia Mason, Seattle, Washington). The two northern‐coastal populations were Metcalf Rd. (Santa Clara County) and Mines Rd. (Alameda County). The two southern/inland populations were from Panoche Hills (Fresno County) and Tesla Rd. (Alameda County). Fresh leaf tissue was collected and kept on ice until it could be homogenized. Cells were lysed in Galbraith’s buffer (Arumuganathan and Earle, 1991) and nuclei were then stained with propidium iodide and treated with RNase. Measurements were performed on a Becton Dickinson FACS flow cytometer (Becton, Dickson and Company, Franklin Lakes, New Jersey). Genome size was estimated in comparison to chicken red blood cells, which were used as an internal standard having been added to the sample before analysis on the flow cytometer (2C = 2.33 pg). Although our choice of standards satisfy several of the recommended criteria for an appropriate size calibration standard (see Table 1 in Suda and Leitch, 2010), it is clear that using animal cells to calibrate plant samples is not ideal (Doležel et al., 2007). Four measurements were taken per sample to determine consistency (2C mean standard error within samples = 0.011 pg [SE within samples ranged from 0.005–0.021]).

**Table 1:** Summary of *trn*L‐F and ITS variable sites among *Caulanthus lasiophyllus* northern/coastal types (c), *C. lasiophyllus* southern/inland types (i), *C. lasiophyllus* putative hybrids (h), *C. flavescens, C. anceps*, and *Streptanthus glandulosus*. Derived states are shaded gray.

	Type	N samples	*trn*L‐F sites			ITS sites											
			111	240	309	118/ 149/ 604	183	250/ 631	251	278	285/ 493	297	494	571	595	606	642
*C. lasiophyllus*	c.1	15	T	G	–	C	C	C	G	C	T	T	G	G	T	–	A
	c.2	6	T	G	–	C	G	C	G	C	T	T	G	G	T	–	A
	c.3	4	T	G	–	C	C	C	G	T	T	T	G	G	T	–	A
	c.4	2	T	G	T	C	C	C	G	C	T	T	G	G	T	–	A
	c.5	1	T	G	T	C	C	C	G	T	T	T	G	G	T	–	A
*C. lasiophyllus*	i.1	12	G	A	–	T	C	C	G	C	C	A	G	T	A	–	C
	i.2	2	G	A	–	T	C	C	G	C	C	A	G	G	A	–	C
	i.3	1	G	A	–	T	C	C	G	C	C	A	G	G	A	T	C
	i.4	6	G	G	–	T	C	C	G	C	C	A	G	G	A	–	C
	i.5	1	T	G	–	T	C	C	G	C	C	A	G	G	A	–	C
*C. lasiophyllus*	h	2	G	G	–	C	C	T	G	C	C	T	G	G	T	–	C
*C. flavescens*		4	T	G	–	C	C	C	A	C	C	T	A	G	T	–	C
*C. anceps*		3	T	G	–	C	C	C	A	C	C	T	G	G	T	–	C
*S. glandulosus*		4	T	G	–	C	C	C	G	T	C	C	G	G	T	–	T

### Morphological analyses

We used digital calipers to measure 13 vegetative and reproductive traits from herbarium specimens (UC & JEPS) related to plant size, leaf size, leaf shape, fruit size, and pubescence of the inflorescences, leaves, and fruits (Table 3). For the quantitative traits, stem length was estimated as the longest distance from the soil surface to the tip of the inflorescence. Stem diameter was measured as the widest point along the stem. The longest leaf was used for measurements of leaf length, leaf width (the maximum width on the longest leaf), petiole length, and leaf sinus depth. Leaf sinus depth was calculated as the difference between the widest point of the leaf and the width of the leaf at the sinus adjacent to the widest point. Fruit length, pedicel length in fruit, and beak length was measured on the longest silique. In addition, four qualitative morphological characteristics were scored for the two types of *C. lasiophyllus* using values between 0–1 in increments of 0.1. The qualitative traits were leaf lobing, stem hairs, leaf hairs, and fruit hairs (all four traits are preceded by “Qual” in Table 3).

For the morphological measurements of the two *C. lasiophyllus* types, the same herbarium samples used in the ITS and *trn*L‐F molecular analyses were included in the morphological analysis. For the few *C. lasiophyllus* samples sequenced from fresh leaf material, we used geographically adjacent herbarium specimens. When necessary, continuous data were natural‐log transformed or square‐root transformed to meet assumptions of normality. Significantly different trait values for comparisons between the two putative types were determined using a series of two‐tailed Student’s t‐tests following Bonferroni correction for multiple testing.

We used two methods to examine overall morphological differentiation between the two lineages—analysis of similarities (ANOSIM; Clarke, 1993) with a nonmetric multidimensional scaling plot (NMDS; Kruskal, 1964) and principal components analysis (PCA) followed by a one‐way analysis of variance (ANOVA). We conducted analysis of similarities (ANOSIM) with Euclidian distances and 9999 permutations to statistically determine if these two lineages represent distinct morphological groupings. Briefly, ANOSIM first calculates a dissimilarity matrix, then uses the rank order of dissimilarity to compare within and between groups producing an R statistic and associated *p*‐value (Clarke, 1993; Warton et al., 2012). *R*‐values greater than zero indicate that dissimilarity between the groups is greater than that within the groups. The statistical significance is determined by comparing the observed *R*‐value to random permutations. We then used the distance matrix from ANOSIM to create a nonmetric multidimensional scaling (NMDS) plot with 500 iterations. We examined the reliability of the agreement between the distance matrix and the ordination using the “stress” metric (Shepard, 1962; Kruskal, 1964). ANOSIM analysis and NMDS plots were done in R 3.4.0 (R Core Team 2017) using the package *vegan* (Oksanen et al., 2019).

In addition, we used a complementary approach, PCA, to visualize the 13 morphological characters in collapsed dimensions and extracted axes for statistical analysis. All characters were z‐score corrected to have a mean of zero and a standard deviation of one. Categorical measures of cauline leaf lobing and pubescence density were included in the PCA because they were based on equidistant categories ranging from 0–1 in increments of 0.1 depending on trichome density—an important trait in Brassicaceae taxonomy. The PCA was conducted in SPSS (v. 18; IBM, Chicago, Illinois, USA) and the first four components collectively explained 73.7% of the total variance. To determine the traits that loaded most heavily on the first four principal component (PC) axes, we used varimax rotation. Differences between the two types of *C. lasiophyllus* in the first four components were examined with ANOVA in Jmp (4.0; SAS Institute, Cary, North Carolina, USA).

We also used PCA to compare *C. lasiophyllus* with its two closest relatives, *C. anceps* and *C. flavescens* (Cacho et al., 2014). The 13 traits measured in *C. lasiophyllus* were recorded for five samples each from *C. anceps* and *C. flavescens* representing their geographic range. Data were transformed and standardized as above and the first three components of the PCA explained 66.9% of the variance. The first four PCs were analyzed with an ANOVA using lineage as the main effect (*C. lasiophyllus* northern/coastal, *C. lasiophyllus* southern‐inland, *C. anceps* or *C. flavescens*). Significant separations in PC values among main effects were identified with Tukey’s HSD test.

Finally, we grew individuals from four populations of *C. lasiophyllus* in the greenhouse to look for morphological characters not detected on herbarium specimens. We digitally scanned the young basal rosette leaves for two to three individuals per population for two populations per type of *C. lasiophyllus*. The two populations representing the southern/inland populations are Tesla Rd. (Alameda County) and Panoche Hills (Fresno County). The two populations representing the northern/coastal populations are Mines Rd. (Alameda County) and Metcalf Rd. (Santa Clara County).

### Climatic niche analysis

In order to examine the climate niches of both types of *C. lasiophyllus*, we compared monthly mean temperature and monthly total precipitation for samples included in the morphological analysis (see above). We extracted PRISM data (30‐year normals from 1981–2010; PRISM Climate Group, 2004) based on each sample’s latitude and longitude (or closest approximation based on locality when latitude and longitude were not available). The two samples from Baja, Mexico were removed because comparable climate data was unavailable from PRISM (*n*_C_ = 24, *n*_I_ = 27). We compared mean annual temperature and total annual precipitation using t‐tests. After recognizing that southern/interior populations appeared to occupy localities with colder winters and hotter summers, we compared “temperature seasonality” as the standard deviation of the 12 monthly mean temperatures per sample with a t‐test. To compare all the climate data in a hypothesis‐testing framework, we used an ANOSIM statistical analysis with an NMDS plot under the same parameters as described for the morphological analysis section.

## RESULTS

### PCR and sequencing

The length of the ITS, *trn*L‐F and *ndh*F loci are consistent with the sizes typical of the Brassicaceae. The ITS region for the *C. lasiophyllus* samples measured 672 bp (GenBank accession numbers JF827154‐JF827215), the *trn*L‐F region measured 685 bp (GenBank accession numbers JF827216‐JF827268), and the *ndh*F region measured 1516 bp (GenBank accession numbers MT548044‐MT548059). We were able to amplify and sequence PCR products from genomic DNA isolated from herbarium specimens as far back as 1935 using standard DNA extraction protocols.

### Sequence analysis

**Figure 2:**
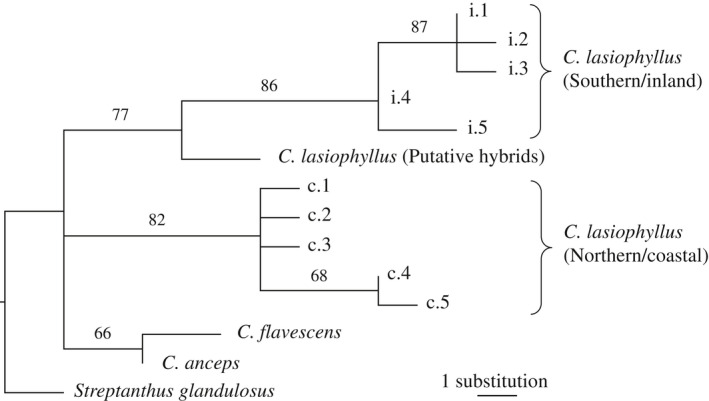
One of 16 equally parsimonious trees using unique haplotypes from the combined ITS, *trn*L‐F, and *ndh*F regions. The unique haplotype number of the northern/coastal type (circles) and southern/inland type (triangles) follow Table 3. Branch length is proportional to the number of substitutions. Bootstrap percentages are indicated above the branches. The tree is rooted with *Streptanthus glandulosus*.

Within *C. lasiophyllus*, we found 14 ITS variable sites and three *trn*L‐F variable sites (Table 1). We also examined *ndh*F for a subset of these *C. lasiophyllus* samples (*N* = 13) and found five additional variable sites (Table 2). Eight ITS variable sites consistently divided 50 out of 52 *C. lasiophyllus* samples into two types (northern/coastal vs. southern/inland; Table 1, Fig. 1). The remaining two samples had a combination of the variable sites for both ITS and trnL‐F (see report of putative hybrids below and in the Discussion). Two of the three *trn*L‐F variable sites differentiated the majority of haplotypes into the two geographically defined lineages. For *trn*L‐F site 111 base pairs (bp) the southern/inland haplotype 5 is shared with the northern/coastal haplotypes and outgroups. At *trn*L‐F site 240 bp, the southern/inland haplotypes 4 and 5 are shared with the northern/coastal haplotypes and outgroups. Three of the five *ndh*F variable sites perfectly differentiated the ITS‐based northern/coastal and southern/inland lineages (Table 2). The other two sites are polymorphic within northern/coastal haplotype 2 (site 694 bp) and unique to *C. flavescens* (site 989 bp; Table 2). Using *Streptanthus glandulosus* as an outgroup to infer the ancestral states of the variable sites, both types of *C. lasiophyllus* are defined by six synapomorphies in a phylogenetic analysis (Fig. 2).

**Table 2:** Variable sites in the chloroplast ndhF gene among *Caulanthus lasiophyllus* northern/coastal types (c), *C. lasiophyllus* southern/inland types (i), *C. lasiophyllus* putative hybrids (h), *C. flavescens, C. anceps*, and *Streptanthus glandulosus*. Derived states are shaded gray.

	Type	N samples	693bp	694bp	965bp	986bp	989bp
*C. lasiophyllus*	c.1	2	T	C	T	A	G
	c.2	3	T	A/C	T	A	G
	c.4	2	T	C	T	A	G
*C. lasiophyllus*	i.1	3	G	A	C	C	G
	i.2	1	G	A	C	C	G
	i.4	1	G	A	C	C	G
*C. lasiophyllus*	h	1	G	A	C	C	G
*C. flavescens*		2	G	A	T	A	C
*C. anceps*		1	G	A	T	A	G
*S. glandulosus*		1	G	A	C	A	G

The ITS sequence divergence within *C. lasiophyllus* is 8× higher than between *C. flavescens* and *C. anceps*. Specifically, there is only one ITS substitution separating *C. flavescens* from *C. anceps*, even though these two species have been long recognized as distinct taxa. There is another single ITS synapomorphy shared by *C. anceps* and *C. flavescens* (site 251 bp) but no synapomorphies uniting the two types of *C. lasiophyllus*. Therefore, even if *C. anceps* and *C. flavescens* are considered a single lineage, there is still 4× more ITS variation between the two *C. lasiophyllus* types than between *C. lasiophyllus* and the lineage formed by *C. anceps* and *C. flavescens*.

Within the two types of *C. lasiophyllus* we also found five subtly distinct haplotypes that differ by a single variable site each except northern/coastal haplotype 5, which has two unique differences. These subgroups follow no clear geographical patterns, yet additional sampling may be warranted to detect any fine scale phylogeographic correlations (Fig. 1).

We applied an approximate molecular clock to the ITS data using an average rate from annual plants (Kay et al., 2006). The mean number of pairwise differences between the northern/coastal and southern/inland haplotypes was 10.8 substitutions (±1.85). Because ITS1 and ITS2 have a length of 508 bp, the divergence equates to 0.0106 substitutions/site. Using the average ITS substitution rate for annual plants of 4.13 × 10^−9^ subs/site/yr (Kay et al., 2006), these two putative lineages split approximately 2.6 million years ago (± 0.44 mya).

The two *C. lasiophyllus* types are differentiated geographically (Fig. 1). Twenty‐eight samples can be unambiguously assigned to the northern/coastal type, which represent 14 northern and coastal counties in California. Within the northern/coastal type there are five haplotypes (based on the ITS and *trn*L‐F data). Northern/coastal haplotype 1 is most common in the San Francisco Bay area ranging south to Monterey County northern/coastal haplotypes 2 and 3 are the only haplotypes in this lineage south of Monterey County, yet can also be found in the San Francisco Bay area and more distantly in the eastern edge of the foothills of the northern Sierra Nevada Mountains (Stanislaus County). Northern/coastal haplotype 4 is known from two samples—one on the western border and another on the eastern border of the southern reach of the San Francisco Bay area (Santa Clara County). Haplotype 5 lies on the border of Santa Clara and Alameda counties.

Another 22 samples belong to the southern/inland lineage, which occurs in 10 primarily inland counties in central and southern California (Fig. 1) extending east to Nevada, Arizona, Sonora (Mexico), and Baja California (Mexico). Haplotype 1 of the southern/inland lineage is the only haplotype that extends north of the Transverse Range. The remaining haplotypes are concentrated in southern California and adjacent states in the United States and Mexico. Haplotype 2 was sampled twice from inland sites—once in northern Inyo County. and another along the southern border of Riverside County. Haplotype 3 is known from a single locality on the northern border of Los Angeles County. Haplotype 4 is the second most common within the southern/inland lineage ranging from Kern County. to northern Baja California and Sonora, Mexico and east to southwestern Nevada. Haplotype 5 was only sampled from southwestern Arizona.

There are two locations where the northern/coastal and southern/inland types occur within 20 km of each other. Both types occur near the San Francisco Bay area, separated by only 7.3 km (Mines Road population and Tesla Road population). Despite the proximity of these two populations, they are in separate watersheds—the Tesla Road population drains west to the inner coast range near Livermore, California and the Corral Hollow Rd. population drains east to the Central Valley near Tracy, California. The other pair of geographically adjacent populations lie along the western edge of the San Joaquin Valley and are separated by approximately 17.1 km. The northern/coastal lineage was sampled from in Piedra Azul Canyon in Merced County (*Taylor12364*) and the southern/inland lineage was sampled “6 mi s Mendota” in Fresno County (*Jepson16986*).

Two *C. lasiophyllus* samples with identical haplotypes exhibit a mosaic of the northern/coastal and southern/inland variable sites for the ITS region (Table 1). Specifically, of the eight sites that differentiate the northern‐coastal and southern‐inland types, the putative hybrid haplotypes have five variable sites in common with the northern‐coastal form (bp 118, 149, 604, 297, and 595) and three variable sites shared with the southern‐inland form (bp 285, 493, and 642). Careful examination of the ITS sequences for these two samples indicate no signs of superimposed nucleotide additivity (Whittall et al., 2000), yet the homogeneous nature of the ITS repeats would need to be confirmed by sequencing >10 clones from individual PCR reactions. These two samples have *trn*L‐F haplotypes that are intermediate between the two types of *C. lasiophyllus* as well (Table 1). At the first variable site (111 bp), the putative hybrid shares the same substitution with 4/5 of the southern/inland haplotypes. At the second variable site (240 bp), the putative hybrid shares the same substitution with all of the northern/coastal types and two of the southern/inland haplotypes. For *ndh*F, the putative hybrid samples share all four differentiating sites with the southern/inland lineage (Table 2).

### Genome sizing

The average genome sizes of northern/coastal and southern/inland types were very similar to one another (northern/coastal: 2C = 0.99 ± 0.029 pg; southern/inland: 2C = 0.95 ± 0.032 pg). In a nonparametric Mann‐Whitney U‐test, the two types are not significantly different (*N*_NC_ = 4, *N*_SI_ = 3; *U* = 9.0; *P* = 0.4), yet this test has limited ability to detect significant differences with such a small sample size.

### Morphological analyses

Although *C. lasiophyllus* exhibits substantial morphological variation across its geographic range, only two of the 13 morphological traits evaluated significantly differentiated the northern/coastal and southern/inland types (Table 3; 10.6084/m9.figshare.12400982). Plants from the northern/coastal lineage had longer fruits (mean ± standard error, 35.06 ± 2.43 mm) compared to the southern/inland lineage (mean ± standard error, 25.37 ± 1.90 mm). The northern/coastal lineage also had significantly longer pedicels in fruit (mean ± standard error, 2.96 ± 0.18 mm) compared to the southern/inland lineage (mean ± standard error, 2.19 ± 0.18 mm). There is still considerable overlap in these two traits, limiting their taxonomic utility.

**Table 3:** Morphological comparison of the two types of *C. lasiophyllus*, *C. anceps*, and *C. flavescens*. Mean (± standard error). Bold values indicate significant differences between the northern/coastal type (*n* = 24) and the southern/inland type (*n* = 27) of *C. lasiophyllus* (Student’s t‐test *p* < 0.05 after Bonferroni correction). Natural log (Ln) and square root (sqrt) transformations were applied.

Species^1^	Ln stem length^2^	Ln stem diameter	Ln leaf length	Ln leaf width	Leaf sinus depth	Sqrt petiole length	Fruit length	Pedicel length in fruit	Beak length	Qual. leaf lobing	Qual. stem hairs	Qual. leaf hairs	Qual. fruit hairs
*C. lasiophyllus* (Northern/coastal)	3.81 (±0.16)	1.05 (±0.10)	4.23 (±0.11)	2.73 (±0.14)	1.66 (±0.15)	4.12 (±0.25)	**35.06** (±2.43)	**2.96** (±0.18)	1.99 (±0.19)	0.75 (±0.09)	0.29 (±0.08)	0.71 (±0.08)	0.18 (±0.07)
*C. lasiophyllus* (Southern/inland)	3.60 (±0.11)	1.01 (±0.10)	4.21 (±0.09)	2.86 (±0.13)	1.84 (±0.13)	3.63 (±0.23)	**25.37** (±1.90)	**2.19** (±0.18)	1.95 (±0.12)	0.59 (±0.08)	0.11 (±0.05)	0.64 (±0.07)	0.18 (±0.06)
*C. anceps*	3.74 (±0.23)	1.15 (±0.20)	4.26 (±0.27)	2.91 (±0.4)	2.65 (±0.32)	3.53 (±0.66)	18.32 (±3.95)	6.19 (±0.43)	2.05 (±0.29)	0.00 (±0.00)	0.00 (±0.00)	0.20 (±0.12)	0.20 (±0.20)
*C. flavescens*	4.06 (±0.11)	1.33 (±0.18)	4.48 (±0.13)	2.98 (±0.17)	2.83 (±0.12)	3.92 (±0.54)	32.46 (±9.76)	6.55 (±0.72)	2.78 (±0.55)	0.05 (±0.05)	0.00 (±0.00)	0.35 (±0.10)	0.45 (±0.23)

^1^ Putative hybrids between the two types of *C. lasiophyllus* were not included in the morphological analysis and statistical tests.

^2^ Stem length in centimeters; all other measurements in millimeters except qualitative measurements (Qual.) that range from 0–1 estimated in intervals of 0.1.

**Figure 3:**
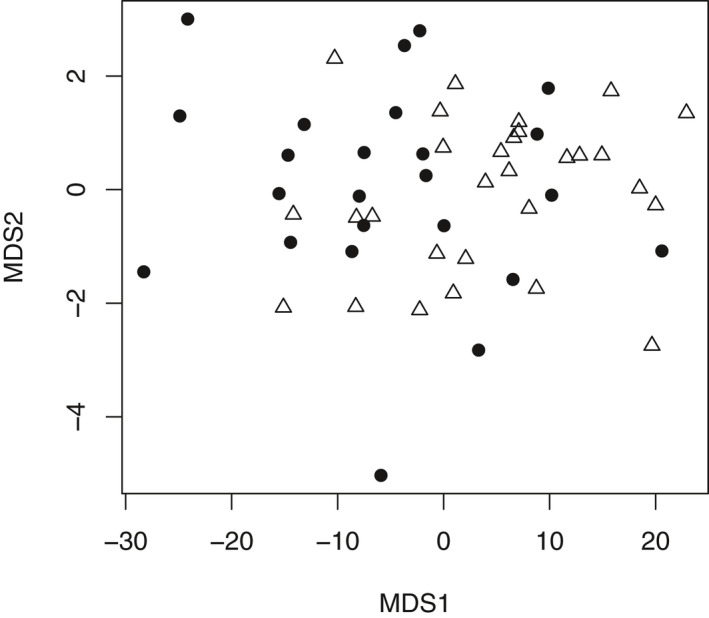
Multidimensional scaling plot for the first two axes representing differences for 13 morphological characters for the two types of *C. lasiophyllus* (filled circles = northern/coastal; open triangles = southern/inland). The minimum stress value (0.01027) indicates excellent representation of the differences in the ordination. The two types are significantly differentiated (ANOSIM, Euclidean distances, 9999 permutations, R statistic = 0.1074, *p* = 0.009).

The ANOSIM analysis and NMDS plot allowed us to use the morphological data to test the molecular hypothesis that there are two lineages within our sampled *C. lasiophyllus*. ANOSIM indicated that the two types were significantly differentiated based on the morphological data (*R* = 0.1074, *P* = 0.009). The NMDS plot using the ANOSIM distance matrix had a very low stress value (0.01027) indicating excellent representation of the differences in the ordination (Oksanen et al., 2019), yet there was still substantial overlap between the northern/coastal and southern/inland types (Fig. 3).

**Figure 4:**
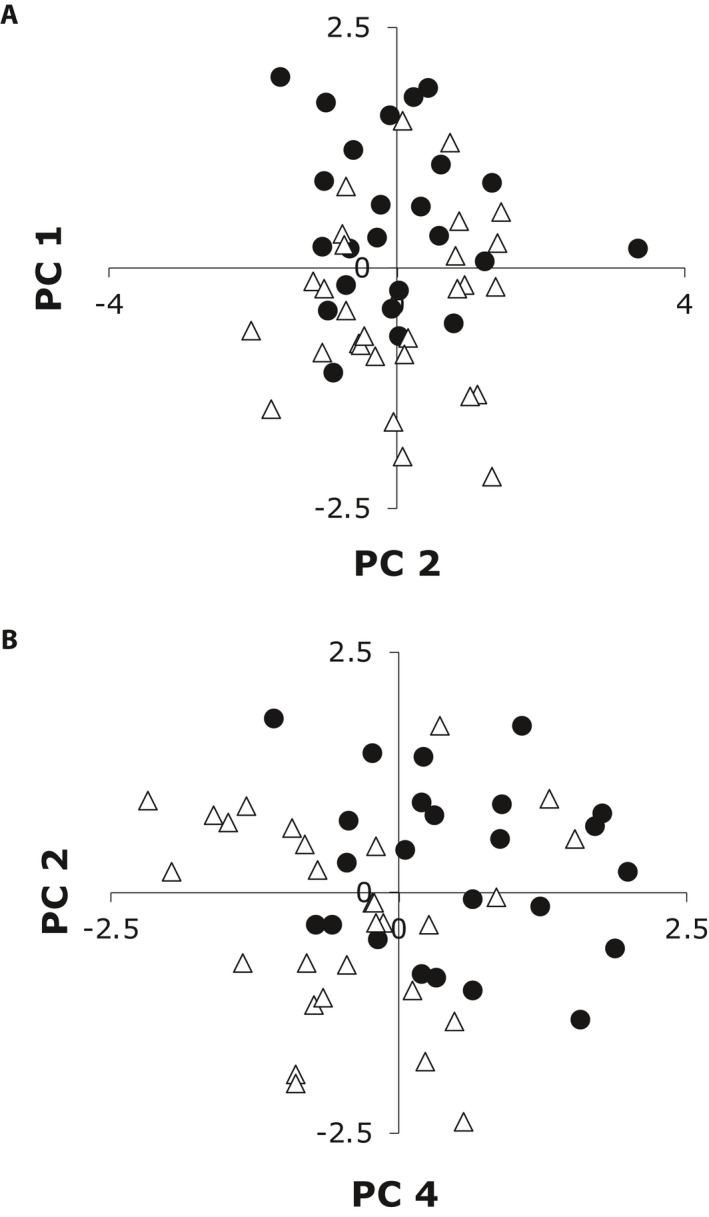
Principal component analysis of 13 morphological traits for the two types of *C. lasiophyllus*. Filled circles are northern/coastal types and triangles are southern/inland types. In (A), principal component axis one (PC1) represents 33.6% of the variance and PC2 explains 14.6% of the variance. In (B), we compare PC2 and PC4, the latter explains an additional 11.7% of the variation. Putative hybrids were not included in the PCA of morphological data because the parental types are not morphologically differentiated.

We conducted two PCAs: one with only *C. lasiophyllus* samples and one with samples from all three species of the “Guillenia Clade.” When focusing on *C. lasiophyllus*, principal component (PC) 1 explains 33.6% of the variation and loads heavily with vegetative traits all in the same direction suggesting an overall size axis (Appendix S1). PC2, which loaded most heavily with the fruit traits that significantly differentiate the two types of *C. lasiophyllus* (Appendix S1), was significantly different between northern/coastal and southern/inland samples (ANOVA: *F*_1,51_ = 13.52, *P* = 0.0006; Fig. 4A); and PC3 did not significantly differentiate the two lineages. There was a weak, yet nonsignificant trend for northern/coastal and southern/inland types to separate along PC4, which loaded heavily with leaf lobing and two pubescence traits (Fig. 4B; Appendix S1; ANOVA: *F*_1,51_ = 3.99, *P* = 0.051). Regardless whether PC2 is compared with PC1 or PC4, there is still considerable overlap of the two lineages (Fig. 4).

When we expand the morphological analysis to also include the closely related *C. anceps* and *C. flavescens*, a similar set of traits load heavily on PC1 (34.6%) suggesting an overall size axis (Appendix S2). PC2 explains 18.3% of the variation, loads heavily with four diverse traits (Appendix S2), and significantly separates the species of the “Guillenia Clade” (ANOVA: *F*_3,59_ = 25.39, *P* < 0.0001; Appendix S3A). Both *C. lasiophyllus* types grouped together and were significantly different from *C. anceps* and *C. flavescens*, which also grouped together (Appendix S3A). There was also differentiation among the species along PC3 (ANOVA: *F*_3,59_ = 3.05, *P* = 0.036), which loaded with several fruit traits, but the post hoc Tukey’s HSD tests found no significant pairwise differences between them (Appendix S3B). Overall, the lack of statistical differentiation between the two *C. lasiophyllus* types, combined with the significant morphological variation among the three species in the “Guillenia Clade,” are consistent with these two types being cryptic species.

**Figure 5:**
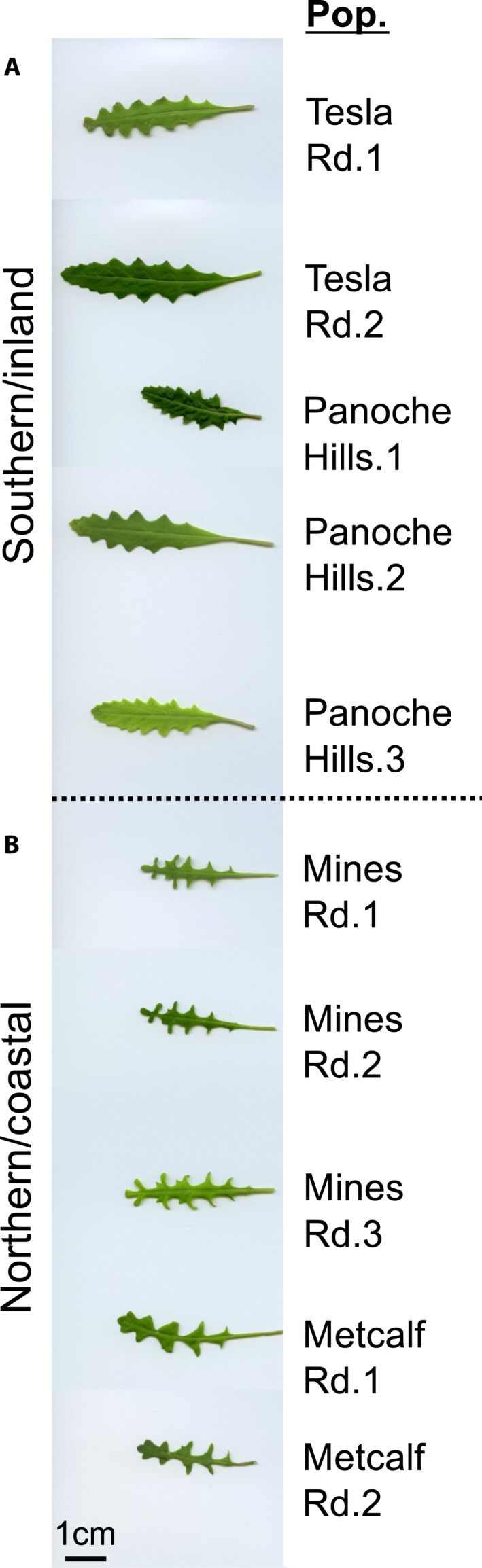
Young basal rosette leaves differentiate the northern/coastal and southern/inland lineages. Each leaf came from a separate individual. Two to three individuals per population for two populations per lineage are shown. Populations are further described in Appendix I.

When growing individuals from four populations of *C. lasiophyllus* in the greenhouse, we documented a single, distinctive, yet short‐lived difference between a limited sampling of the two types of *C. lasiophyllus* in the first leaves of the basal rosette (Fig. 5). Individuals from the southern/inland lineage had leaf margins with considerably shallower lobes compared to the deep and wide pinnatifid lobing of the northern/coastal lineage.

### Climatic niche analysis

Both mean annual temperature and total annual precipitation are significantly different between the two types of *C. lasiophyllus* (10.6084/m9.figshare.12400985). The total annual precipitation is 2.2× higher in the northern/coastal type compared to the southern/inland type (ttest, *p* = 2×10^−7^; Appendix S4A). The mean annual temperature is just 8% higher for the southern/interior samples compared to the northern/coastal samples (ttest, *p* = 0.021; Appendix S4B), yet the “temperature seasonality” (average standard deviation of monthly mean temperatures) is 34% higher among the southern/inland samples compared to the northern/coastal samples (ttest, *p* = 2.2 × 10^−8^; Appendix S4B).

**Figure 6:**
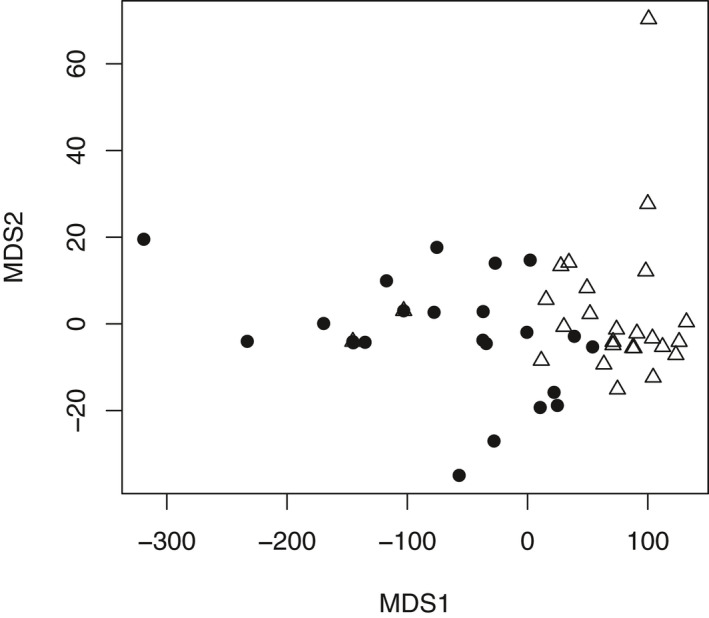
Multidimensional scaling plot for the first two axes representing differences in 30‐year normals for mean monthly temperature and total monthly precipitation between the two types of *C. lasiophyllus* (filled circles = northern/coastal; open triangles = southern/inland). Two samples from Baja, Mexico were not included because of lack of PRISM data. The minimum stress value (0.01738819) indicates excellent representation of the differences in the ordination. The two types are significantly differentiated (ANOSIM, Euclidean distances, 9999 permutations, R statistic = 0.4178, *p* < 0.0001).

The overall climatic niche based on 30‐year normals for monthly mean temperature and monthly total precipitation (24 variables for 51 samples) are significantly different for the two types of *C. lasiophyllus* (ANOSIM, 9999 permutations, *R* = 0.4178, *p* < 0.0001). The NMDS plot shows strong differentiation of the two types (Fig. 6) with a very low stress value (0.01027) indicating excellent representation of the differences in the ordination (Oksanen et al., 2019). To determine the relative roles of temperature and precipitation, we ran separate ANOSIM analyses and found that precipitation differentiated the two types of *C. lasiophyllus* samples ~2× better than temperature (*R*_precip_ = 0.4164, *p* < 0.0001; *R*_temp_ = 0.2051, *p* < 0.0001).

## DISCUSSION

### Cryptic species

This study has uncovered two molecularly distinct, putatively independent lineages within *C. lasiophyllus* that are several times more molecularly divergent than the other two morphologically unique species in the “Guillenia Clade.” At the outset, we note that the two putative lineages we detected are based on thorough, yet not exhaustive, biogeographic sampling and only small portions of the nuclear and chloroplast genomes. Additional samples from the extremes of the edges of the range of *C. lasiophyllus*, especially northwestern and southeastern California would be necessary to confirm the biogeographic divergence described herein. Furthermore, more genome coverage, especially of the nuclear genome, with rapidly evolving regions (e.g., microsatellites) or a more genome‐scale approach (e.g., RAD‐Seq) could also be done to confirm the molecular distinctiveness of these two putative lineages. Recent genome‐scale investigations in *Mimulus* have detected cryptic lineages of perennial *M. decorus* that exhibits hybrid seed inviability (Coughlan et al., 2020) that would be interesting to test for in *C. lasiophyllus*.

The two potential cryptic lineages of *C. lasiophyllus* can be divided geographically into northern/coastal and southern/inland areas. These two lineages do not coincide with the distributions of any of the three previously defined varieties of *C. lasiophyllus*, nor combinations thereof (Payson, 1922; Jepson, 1936; Munz, 1959). Jepson (1936) treats *C. l*. var. *inalienum* as occurring from Solano County to San Luis Obispo County, which overlaps with the northern/coastal lineage, but neglects to include Marin County to the north and several coastal counties to the south (Santa Barbara, Los Angeles, and San Diego counties; Fig. 1). Similarly, *C. lasiophyllus* var. *rigidum* is expected from Glenn County to eastern Contra Costa County, yet also neglects Marin County to the north and numerous coastal counties to the south (Fig. 1). Even when these two varieties are treated together, they are still inadequate at describing the distribution of either of the two *C. lasiophyllus* cryptic lineages identified herein. The third variety, *C. lasiophyllus* var. *utahensis*, applies to *C. lasiophyllus* from the Colorado and Mohave deserts in California and adjacent regions (Nevada, Arizona, Colorado, and Utah; Payson, 1922; Jepson, 1936). Although all our samples from the California deserts and neighboring states belong to the southern/inland lineage, this variety fails to account for many nondesert counties where the southern/inland lineage is also found (eastern Contra Costa County, San Benito County, Fresno County, Kern County, eastern San Luis Obispo County, and Ventura County.; Fig. 1). Furthermore, none of these previously described varieties coincide with the haplotypes within each cryptic lineage because these haplotypes do not exhibit a strong geographic pattern (Fig. 1).

Not only are these two putative lineages inconsistent with the geographic distribution of previously described varieties, they are also largely morphologically indistinguishable from one another. Our morphometric analysis of 13 of the morphological traits that frequently appear in keys to these taxa revealed only two statistically significant differences (fruit length and pedicel length in fruit) and even these traits have substantial overlap in their range of values. The previously described varieties of *C. lasiophyllus* have been differentiated by (1) the angle of the fruit (erect vs. deflexed), which we have observed as variable within populations and even within individuals as they develop (J. B. Whittall, Santa Clara University, unpublished data), and (2) stem pubescence (glabrous vs. densely hairy), which we evaluated yet fails to differentiate these cryptic lineages (Table 3). When all 13 traits are considered together, PC2 loads heavily for the two differentiating characteristics and statistically separates the two cryptic lineages, but with considerable overlap (Fig. 4). Other studies of cryptic species that include extensive morphological analyses have reported few or no differentiating characters in both plants (Carter, 2012) and animals (Rawsey and Egge, 2017).

While growing individuals of *C. lasiophyllus* in the greenhouse, we documented a single, short‐lived morphological difference between a limited sampling of the two cryptic lineages in the first leaves of the basal rosette. Although we did not detect any differences in the leaf sinus depth in our morphometric study of herbarium specimens, we measured only the cauline leaves because the basal rosette is typically withered away by the time the specimens were collected in flower and/or fruit. This finding suggests that further investigation into additional morphological characteristics (e.g., seeds) and more physiologically relevant traits may yield additional distinguishing characteristics. This is especially relevant given the climatic differences associated with the distributions of the two cryptic lineages. Similar physiological differentiation was suggested by Clausen (1951) in reference to the lack of morphologically distinct characteristics in cryptic species along environmental gradients in California.

### Biogeography

The geographic division within *C. lasiophyllus* reported here is unique among a diversity of California plant and animal phylogeographic studies (Shaw, 2000; Chan et al., 2001; Carlsbeek et al., 2003; Angert and Schemske, 2005; Baldwin, 2006; LaPointe and Rissler, 2006; Crummett and Eernisse, 2007; Graves and Schrader, 2008; Baunsteiger et al., 2012; Carter, 2012; Yost et al., 2012; Highton, 2014; Reilly and Wake, 2015; Emata and Hedin, 2016). Three common biogeographic barriers in California are the Central Valley (separating the Coast Ranges from the Sierra Nevada), the Transverse Range (east–west mountains separating Northern and Southern California), and the San Francisco and Monterey bays (separating the north and central coasts). Although the Central Valley is an important barrier for many plant and animal lineages (Carlsbeek et al., 2003; Baldwin, 2006; Emata and Hedin, 2016), *C. lasiophyllus* was found historically at numerous stations across the Central Valley. Furthermore, it is relatively rare to the east of the Central Valley (restricted to the low elevations in the foothills of the Sierra Nevada). Therefore, it was not surprising that there is no clear phylogeographic split between the coast range populations and those from the foothills of the Sierra Nevada (Fig. 1), unlike that found in the northern half of the Central Valley separating the Coastal Clade from the Northern Clade in a genome‐wide phylogeographic study of *Mimulus guttatus* (Twyford and Friedman, 2015).

Another frequently detected phylogeographic barrier in California is the Transverse Range (Carlsbeek et al., 2003; Lapointe and Rissler, 2005). The Transverse Range does not appear to be a current barrier for *C. lasiophyllus* because both cryptic lineages can be found both north and south of it (Fig. 1). Instead, as one progresses southward, the northern/coastal lineage becomes increasingly restricted to the coast (Santa Barbara County. southward) with only two northern‐coastal haplotypes represented (haplotypes 2 and 3). Similarly, only one haplotype (1) from the southern‐inland lineage is present north of the Transverse Range (Fig. 1); also similarly, the Transverse Range was not a phylogeographic barrier after examining whole genome sequences for *Mimulus guttatus* within the Southern Clade (Twyford and Friedman, 2015).

Numerous studies have also documented strong phylogeographic barriers around the San Francisco and Monterey Bay areas in plants (Graves and Schrader, 2008; Yost et al., 2012) and animals (Carlsbeek et al., 2003), especially salamanders (Reilly and Wake, 2015). Although both cryptic lineages of *C. lasiophyllus* can be found in the greater San Francisco Bay area (Fig. 1), they do not reflect the phylogeographic divisions like those found in *Dirca occidentalis* between East Bay and San Francisco Peninsula populations (Graves and Schrader, 2008). In the southern and eastern portions of the San Francisco Bay area, there is a remarkable concentration of all five haplotypes of the northern/coastal lineage. An analogous biogeographic pattern of northern/coastal versus southern/inland exists in *Mimulus guttatus* (Twyford and Friedman, 2015) and *Polystichum munitum*, but both species have distributions extending much farther northward through the Pacific Northwest (Soltis et al., 1997). In both cases, the pattern at the northern edge of these two species’ ranges are attributed to postglacial recolonization either from opposing directions (northern versus southern refugia) or from a polymorphic southern refugium, that may apply to *Caulanthus lasiophyllus* outside of California where we focused our sampling.

While our two putative cryptic lineages were not divided by traditionally recognized biogeographic barriers, they were strongly separated by climate, suggesting a possible driver of divergence in these two groups. The northern‐coastal lineage was found in wetter, less seasonal, and somewhat cooler environments compared to the southern‐inland lineage.

If the concentration of haplotypes within each lineage reflects their historical centers of diversity, then the northern‐coastal lineage of *C. lasiophyllus* would be centered in the San Francisco Bay area. Meanwhile, the southern‐inland lineage would be centered in southern California with four of the five haplotypes concentrated in inland regions (Fig. 1). In fact, only haplotype 1 of the southern‐coastal lineage was sampled north of the Transverse Range where the frequency of the southern‐inland lineage is much less common (6 out of 22 samples). Similarly, for the northern‐coastal type only two of the five haplotypes can be found south of the Transverse Range (haplotypes 2 and 3) and it is relatively rare there as well (6 out of 28 northern/coastal samples are south of the Transverse Range).

To help interpret the geological setting when these two lineages diverged, we applied an approximate molecular clock for the ITS region of annual plants (Kay et al., 2006). Using the average ITS substitution rate for annual plants (Kay et al., 2006), we estimate these two putative lineages split approximately two to three million years ago. This coincides with the final stage in the development of the Coast Range and the Transverse Range (5–2 mya) (Chamberlain and Poage, 2000) and changes in Pacific Ocean currents leading to increasing aridity in California (Ravelo et al., 1997). Our molecular clock analyses suggest that the two cryptic lineages we describe diverged during a time when the Monterey Bay was the principal outlet for a great inland sea and subsequently a lake that filled today’s Central Valley (3.3–0.6 mya; Raven and Axelrod, 1978; Huber, 1981; Unruh, 1991; Chamberlin and Poage, 2000), yet we see no strong footprint of this past phylogeographic barrier in *C. lasiophyllus* (Fig. 1). In light of the geological history during the origin of these cryptic species, we envision an original splitting event of the two lineages on either side of the Transverse Range followed by subsequent dispersal of a subset of the haplotypes into each other’s original geographic range.

### Putative hybrids

The molecular distinctiveness of these two lineages suggests they may be reproductively isolated. Unfortunately, attempts at directly testing for postpollination barriers to gene flow were stymied by the discovery that pollen is dehisced atop receptive stigmas in the flower buds of this obligate selfer (J. B. Whittall, Santa Clara University, unpublished data). Without crossing results, no final conclusions can be drawn regarding reproductive isolation, yet two contradictory observations on the potential for hybridization should be mentioned.

First, we documented two putative hybrids based on chimeric ITS sequences. Although these could be the product of alternative gene conversion or concerted evolution from a polymorphic ancestral sequence, the degree of ancestral polymorphism in ITS required to produce these chimeric sequences would be unprecedented (Small et al., 2004). If instead these are hybrid individuals, the lack of superimposed nucleotide additivity patterns suggests these putative hybrids have not undergone complete concerted evolution or gene conversion (Whittall et al., 2000). In fact, both putative hybrid samples share two unique ITS variable sites (Table 1). We suggest that they represent one or more historical hybridization event(s) between the two cryptic lineages followed by concerted evolution to homogenize the ITS repeats into a chimera of the two geographically defined lineages (Small et al., 2004; Campbell et al., 1997).

These putative hybrid individuals have more in common with the southern/inland lineage than the northern/coastal lineage. They share haplotypes with the southern/inland lineage at 50% of the *trn*L‐F variable sites (Table 1) and 100% of the *ndh*F variable sites (Table 2). They are also morphologically aligned with the southern/inland lineage (fruit lengths of 24.3 mm and 18.8 mm and pedicel lengths of 1.31 mm and 1.41 mm; compare to Table 3). Furthermore, these two samples are geographically allied with the southern/inland lineage (Fig. 1), yet both are from Central Valley Grasslands, which represents an unusual habitat for both *C. lasiophyllus* cryptic lineages. The samples are from Kesterson National Wildlife Refuge in Merced County. and near the Carrizo Plain in San Luis Obispo County. Multiple attempts at relocating the Kesterson population in 2008 were unsuccessful, but these surveys confirmed the unexpected habitat for the species. Overall, these two putative hybrid samples suggest some level of (historical) gene flow between the two lineages.

Second, in contrast, it is noteworthy that for at least two locations, the two lineages can be found very close together (Fig. 1). In one circumstance, there are two populations representing the two cryptic lineages that are 7 km separated. In another instance, the two lineages are 17 km apart. Yet, in both cases the two cryptic lineages remain molecularly and morphologically distinct based on ITS sequences (Table 1), *ndh*F sequences (Table 2) and in their degree of lobing in juvenile basal rosette leaves (Fig. 5). Additional surveys in the eastern portion of the San Francisco Bay area (eastern Alameda County) and along the western edge of the San Joaquin Valley (at the confluence of San Benito, Merced, and Fresno counties; see arrows in Fig. 1) are necessary to confirm the lack of hybrids and help identify additional distinguishing ecological or physiological differences. This observation suggests some degree of reproductive isolation between the two cryptic lineages, contrary to the existence of putative hybrids.

### Genome sizing

A comparison of genome sizes in two populations of each cryptic lineage indicates no consistent differences. Our mean *C. lasiophyllus* genome size (1C = 0.49) was 0.11 pg larger than previously reported by Johnston et al. (2005; 1C = 0.38 pg under the synonym *Guillenia lasiophylla*). Our estimate is very similar to the ancestral state for the Brassicaceae (1C = 0.50 pg; Lysak et al., 2008), yet three times larger than that of *Arabidopsis thaliana* (1C = 0.16 pg; Bennett et al., 2003), one of the smallest genomes in flowering plants (Lysak et al., 2008). Within the Thelypodieae (syn. Schizopetaleae), *C. lasiophyllus* is one of the smallest genomes estimated to date at 71% the size of *Streptanthus polygaloides* (1C = 0.69) and similar in size to *Sisymbrium* (Lysak et al., 2008). The relatively small genome of *C. lasiophyllus* (along with *C. amplexicaulis* var. *barbarae*; Lysak et al., 2008), rapid growth rate, availability of microsatellite primers in close relatives (Burrell and Pepper, 2006), and autogamous mating system may facilitate further studies of the evolutionary origins of cryptic species.

## CONCLUSIONS

Fifty‐two individuals from across California were sequenced for two rapidly evolving loci representing the nuclear (ITS) and chloroplast (*trn*L‐F) genomes. A subset of the samples was also sequenced for a second chloroplast locus (*ndh*F). Two evolutionarily distinct, climatically differentiated, yet morphologically cryptic lineages emerged. These two lineages are geographically distinct (northern‐coastal vs. southern‐inland), yet do not follow the distributions of the three previously described varieties of *C. lasiophyllus*. The two lineages only differ in two morphological traits (yet are broadly overlapping) and potentially in the degree of pinnatifid divisions in the young basal rosette leaves. Reproductive isolation is unknown, but the identification of two putative hybrid samples raises the possibility of contemporary gene flow between geographically adjacent populations of the two cryptic lineages. Based on genome sizing, ploidy differences are unlikely a reproductive isolating mechanism. Carefully conducted crosses between the two lineages and additional field surveys in the areas where putative hybrids have been documented, or are likely to occur, are necessary to confirm complete reproductive isolation of these two lineages. Further investigations into any physiological differences between the two types could link the climatic niche differentiation with the molecular distinctiveness and potentially identify additional discriminating characteristics.

## AUTHOR CONTRIBUTIONS

J.W., T.B., and C.D. contributed to the conception and design of the study and the data acquisition. J.W., T.B., C.D., and B.S. conducted the analyses and interpreted the data. J.W., T.B., C.D., and B.S. were involved in drafting and revising the manuscript.

## Supporting information


**APPENDIX S1.** Principal component (PC) axis loadings of morphological traits using only *Caulanthus lasiophyllus* samples. The most substantial loadings for each axis are indicated in bold. “Qual” indicates a qualitative estimate ranging from 0–1 in categories of 0.1. Natural log (Ln) and square root (sqrt) transformations were applied.

**APPENDIX S2.** Principal component (PC) axis loadings of morphological traits measured in *Caulanthus lasiophyllus*, *C. anceps*, and *C. flavescens* samples. The most substantial loadings for each axis are indicated in bold. “Qual” indicates a qualitative estimate ranging from 0–1 in categories of 0.1.

**APPENDIX S3.** Principal components analysis of 13 morphological traits for both *C. lasiophyllus* lineages, *C. flavescens*, and *C. anceps*. In (A), principal component axis one (PC1) and PC2 explain 34.6% and 18.3% of the variation, respectively. In (B), PC2 and PC3 are compared. PC3 explains an additional 14.0% of the variation.

**APPENDIX S4.** Climate niche data for two types of *C. lasiophyllus* samples. Violin plots for the northern/coastal samples (gray fill) and southern/inland samples (no fill) are from PRISM’s 30 year normals (1981–2010). Medians are indicated with black circles. First through third quartiles are indicated with thickened black, vertical lines. Months are listed in calendar order abbreviated by their first letter followed by annual values. (A) Total precipitation by month and for the annual total (Ann). Note: separate *y*‐axis for annual total on right. (B) Mean temperature by month and for the annual total (Ann).

## Data Availability

All sequences generated in this study are deposited in GenBank (Appendix S1). Morphological data is available at FigShare (“caulanthus lasiophyllus data for ANOSIM.tab delimited.txt”; https://doi.org/10.6084/m9.figshare.12400982.v1). Climate data used in this study are also available at FigShare (“Precipitation and Temperature Data for *Caulanthus lasiophyllus*”; https://doi.org/10.6084/m9.figshare.12400985.v1).

## Appendix 1 Localities, haplotype assignment, GenBank accession numbers, voucher specimens for samples included in the molecular study. c = northern‐coastal lineage; i = southern‐inland lineage; h = putative hybrid samples. Blank cells for GenBank accessions represent missing data.

**Table:**

Taxon	Collection locale	Haplotype	ITS GenBank Accession	*trn*L‐F GenBank Accession	Voucher specimen
*C. lasiophyllus*	USA: Silver Creek Hills, Santa Clara Co., CA	c.1	JF827155		SJSU1637
*C. lasiophyllus*	USA: Santa Clara Co., CA	c.1		JF827216	Whittall 13498
*C. lasiophyllus*	USA: Roosevelt Creek, Junipero Serro Park, Monterey Co., CA	c.1	JF827169	JF827227	JEPS74254
*C. lasiophyllus*	USA: Santa Clara Co.	c.1		JF827217	Whittall 13555
*C. lasiophyllus*	USA: Monticello Dam, Vaca Mountains, Napa Co., CA	c.1	JF827171	JF827229	JEPS106824
*C. lasiophyllus*	USA: American Canyon, Avilla Ranch, Napa Co., CA	c.1	JF827172	JF827230	JEPS106784
*C. lasiophyllus*	USA: Metcalf Rd., Santa Clara Co., CA	c.1	JF827160	JF827218	Whittall 13674
*C. lasiophyllus*	USA: Santa Clara Co., CA	c.1		JF827220	Whittall 12998
*C. lasiophyllus*	USA: Sulfur Spring, Mount Vallejo, Solano Co., CA	c.1	JF827175	JF827234	JEPS108123
*C. lasiophyllus*	USA: Santa Clara Co., CA	c.1	JF827162	JF827221	Whittall 12976
*C. lasiophyllus*	USA: Mix Canyon, Vaca Mountains, Solano Co., CA	c.1	JF827176	JF827235	JEPS84644
*C. lasiophyllus*	USA: Santa Clara Co., CA	c.1	JF827161	JF827219	Whittall 14674
*C. lasiophyllus*	USA: Piedra Azul Canyon, Merced Co., CA	c.1	JF827191	JF827249	UC1736547
*C. lasiophyllus*	USA: Putah Creek near Rte. 128, Solano Co., CA	c.1	JF827199	JF827257	UC1585848
*C. lasiophyllus*	USA: West of Paskenta, Tehema Co., CA	c.1	JF827200	JF827258	UC1002631
*C. lasiophyllus*	USA: Canada Lobos, Santa Rosa Island, Santa Barbara Co., CA	c.2		JF827232	JEPS93788
*C. lasiophyllus*	USA: San Miguel Island, Santa Barbara Co., CA	c.2	JF827174	JF827233	JEPS96334
*C. lasiophyllus*	USA: San Miguel Island, Santa Barbara Co., CA	c.2	JF827156	JF827223	Whittall 13876
*C. lasiophyllus*	USA: Santa Clara Co., CA	c.2	JF827163	JF827222	Whittall 13888
*C. lasiophyllus*	USA: Crestridge Ecological Reserve, San Diego Co., CA	c.2	JF827193	JF827251	UC1790110
*C. lasiophyllus*	USA: Viejas Mountain, San Diego Co., CA	c.2	JF827194	JF827252	UC1790171
*C. lasiophyllus*	USA: Jamison Creek, Santa Cruz Co., CA	c.2	JF827198	JF827256	UC1023224
*C. lasiophyllus*	USA: Las Flores Canyon, Los Angeles Co., CA	c.3	JF827170	JF827228	JEPS18470
*C. lasiophyllus*	USA: Mount Diablo, Contra Costa Co., CA	c.3	JF827181	JF827239	UC691080
*C. lasiophyllus*	USA: Santa Cruz Island, Santa Barbara Co., CA	c.3	JF827197	JF827255	UC1558085
*C. lasiophyllus*	USA: Senter Road Hills, Santa Clara Co., CA	c.4	JF827154		SJSU1637
*C. lasiophyllus*	USA: Mines Road, Alameda Co., CA	c.4	JF827164		Whittall 13455
*C. lasiophyllus*	USA: Alameda Co., CA	c.5	JF827179	JF827238	UC1607642
*C. lasiophyllus*	USA: South Mendota, Fresno Co., CA	i.1	JF827166	JF827224	JEPS54074
*C. lasiophyllus*	USA: Tesla Road, Alameda Co., CA	i.1	JF827165		Whittall 14001
*C. lasiophyllus*	USA: Edwards Airforce Base, Kern Co., CA	i.1	JF827168	JF827226	JEPS84981
*C. lasiophyllus*	USA: Calico Mountains, San Bernadino Co., CA	i.1	JF827173	JF827231	JEPS54073
*C. lasiophyllus*	USA: Cuyama River, Ventura Co., CA	i.1	JF827178	JF827237	JEPS27768
*C. lasiophyllus*	USA: Corral Hollow, Alameda Co., CA	i.1	JF827180		UC1057468
*C. lasiophyllus*	USA: Panoche Hills, Fresno Co., CA	i.1	JF827159	JF827240	UC1561436
*C. lasiophyllus*	USA: Nelson Range, Saline Valley, Inyo Co., CA	i.1	JF827183	JF827242	UC1534994
*C. lasiophyllus*	USA: Mule Spring, Inyo Mountains, Inyo Co., CA	i.1	JF827185	JF827244	UC1736548
*C. lasiophyllus*	USA: Adobe Mountain, Los Angeles Co., CA	i.1	JF827189	JF827247	UC1595468
*C. lasiophyllus*	USA: Soda Lake, San Luis Obispo Co., CA	i.1	JF827196	JF827254	UC1296267
*C. lasiophyllus*	USA: Corral Hollow, Alameda Co., CA	i.1	JF827202	JF827259	UC1084429
*C. lasiophyllus*	USA: Wilkerson Springs, Inyo Co., CA	i.2	JF827184	JF827243	UC1535191
*C. lasiophyllus*	USA: Vail Lake, Riverside Co., CA	i.2	JF827192	JF827250	UC1584126
*C. lasiophyllus*	USA: San Mateo Canyon, San Diego Co., CA	i.2	JF827158		UC1608301
*C. lasiophyllus*	USA: La Grange, Stanislaus Co., CA	i.2	JF827177	JF827236	JEPS45388
*C. lasiophyllus*	USA: Soledad Mountain, Kern Co., CA	i.3	JF827167	JF827225	JEPS29326
*C. lasiophyllus*	USA: Jacumba Natural Area, Imperial Co., CA	i.4	JF827182	JF827241	UC1787778
*C. lasiophyllus*	USA: Red Rock Canyon, Kern Co., CA	i.4	JF827186		UC1096404
*C. lasiophyllus*	USA: Isabelle Lake, Kern Co., CA	i.4	JF827187	JF827245	UC1561855
*C. lasiophyllus*	USA: Hwy 138/Hwy 18, Los Angeles Co., CA	i.4	JF827188	JF827246	UC1563060
*C. lasiophyllus*	USA: Searchlite, Clark County, NV	i.4	JF827203	JF827260	UC048030
*C. lasiophyllus*	Mexico: Baja California	i.4	JF827204		UC1605415
*C. lasiophyllus*	Mexico: Baja California	i.4	JF827205	JF827261	UC1351153
*C. lasiophyllus*	USA: South of Ajo, Pimo Co., AZ	i.4	JF827207	JF827263	UC1353394
*C. lasiophyllus*	USA: Salt River Mountains, Maricopa Co., AZ	i.5	JF827206	JF827262	UC489453
*C. lasiophyllus*	USA: Kesterson National Wildlife Refuge, Merced Co., CA	h	JF827190	JF827248	UC1558004
*C. lasiophyllus*	USA: Cholame Valley, San Luis Obispo Co., CA	h	JF827195	JF827253	UC1296266
*C. flavescens*	USA: Corral Hollow, Alameda Co., CA	NA		JF827267	JEPS87015
*C. flavescens*	USA: North of Gonzales, Monterey Co., CA	NA		JF827266	UC1736546
*C. flavescens*	USA: Mount Diablo, Contra Costa Co., CA	NA	JF827212		Whittall 14030
*C. flavescens*	USA: Mount Diablo, Contra Costa Co., CA	NA	JF827211		UC1736552
*C. flavescens*	USA: Hernandez River, San Benito Co., CA	NA		JF827268	JEPS105326
*C. anceps*	USA: Shandon‐Bitterwater Road, San Luis Obispo Co., CA	NA	JF827214	JF827265	UC1529013
*C. anceps*	USA: Potrero Sierra Madre Mountains, Santa Barbara Co., CA	NA	JF827215	JF827264	UC1488859
*C. anceps*	USA: Griswold Hills, San Benito Co., CA	NA	JF827213		UC1736551
*Streptanthus glandulosus*	USA: Santa Clara Co., CA	NA	DQ829804, DQ829785, AF346651, DQ829798		
