# Supplementary material for: Two cryptic species of California mustard within Caulanthus lasiophyllus
Source: Am J Bot. 2020 Dec 28;107(12):1815–30. doi: 10.1002/ajb2.1562 (PMC7839454; doi:10.1002/ajb2.1562)
Supplement: Supplementary file 1 — APPENDIX S1. Principal component (PC) axis loadings of morphological traits using only Caulanthus lasiophyllus samples. The most substantial loadings for each axis are indicated in bold. “Qual” indicates a qualitative estimate ranging from 0–1 in categories of 0.1. Natural log (Ln) and square root (sqrt) transformations were applied. [file AJB2-107-1815-s001.docx]

Appendix S1. Principal component (PC) axis loadings of morphological traits using only *Caulanthus lasiophyllus* samples. The most substantial loadings for each axis are indicated in bold. “Qual” indicates a qualitative estimate ranging from 0-1 in categories of 0.1. Natural log (Ln) and square root (sqrt) transformations were applied.

|  | Rotated Component Loadings | | | |
| --- | --- | --- | --- | --- |
| Morphological Trait | PC1 (33.6%) | PC2 (14.6%) | PC3 (13.8%) | PC4 (11.7%) |
| Ln stem length | **0.651** | 0.470 | -0.069 | -0.094 |
| Ln stem diameter | **0.806** | 0.305 | -0.214 | -0.013 |
| Ln leaf length | **0.922** | 0.136 | -0.084 | -0.095 |
| Ln leaf width | **0.934** | 0.013 | -0.167 | -0.012 |
| Leaf sinus depth | **0.784** | -0.031 | -0.162 | -0.461 |
| Sqrt petiole length | **0.768** | 0.165 | -0.092 | 0.098 |
| Fruit length | 0.201 | **0.887** | 0.200 | -0.051 |
| Pedicel length | 0.122 | **0.852** | -0.206 | 0.029 |
| Beak length | -0.040 | 0.076 | **0.839** | -0.147 |
| Qual. leaf lobing | 0.219 | -0.038 | -0.006 | **0.784** |
| Qual. stem hairs | -0.213 | -0.018 | **0.526** | **0.569** |
| Qual. leaf hairs | -0.351 | 0.006 | -0.116 | **0.552** |
| Qual. fruit hairs | -0.314 | -0.147 | **0.777** | 0.131 |
