# Supplementary material for: Two cryptic species of California mustard within Caulanthus lasiophyllus
Source: Am J Bot. 2020 Dec 28;107(12):1815–30. doi: 10.1002/ajb2.1562 (PMC7839454; doi:10.1002/ajb2.1562)
Supplement: Supplementary file 2 — APPENDIX S2. Principal component (PC) axis loadings of morphological traits measured in Caulanthus lasiophyllus, C. anceps, and C. flavescens samples. The most substantial loadings for each axis are indicated in bold. “Qual” indicates a qualitative estimate ranging from 0–1 in categories of 0.1. [file AJB2-107-1815-s002.docx]

Appendix S2. Principal component (PC) axis loadings of morphological traits measured in *Caulanthus lasiophyllus*, *C. anceps* and *C. flavescens* samples. The most substantial loadings for each axis are indicated in bold. “Qual” indicates a qualitative estimate ranging from 0-1 in categories of 0.1.

|  | Rotated Component Loadings | | |
| --- | --- | --- | --- |
| Morphological Trait | PC1 (34.6%) | PC2 (18.3%) | PC3 (14.0%) |
| Ln stem length | **0.793** | –0.152 | 0.087 |
| Ln stem diameter | **0.863** | –0.168 | –0.097 |
| Ln leaf length | **0.893** | –0.213 | –0.087 |
| Ln leaf width | **0.876** | –0.139 | –0.208 |
| Leaf sinus depth | **0.617** | **–0.659** | –0.152 |
| Sqrt petiole length | **0.784** | –0.003 | –0.034 |
| Fruit length | **0.511** | 0.089 | **0.562** |
| Pedicel length | 0.222 | **–0.641** | 0.309 |
| Beak length | –0.020 | –0.164 | **0.794** |
| Qual. leaf lobing | 0.204 | **0.812** | –0.088 |
| Qual. stem hairs | –0.212 | 0.555 | 0.429 |
| Qual. leaf hairs | –0.208 | **0.639** | 0.039 |
| Qual. fruit hairs | –0.342 | 0.046 | **0.700** |
