# Supplementary material for: Two cryptic species of California mustard within Caulanthus lasiophyllus
Source: Am J Bot. 2020 Dec 28;107(12):1815–30. doi: 10.1002/ajb2.1562 (PMC7839454; doi:10.1002/ajb2.1562)
Supplement: Supplementary file 3 — APPENDIX S3. Principal components analysis of 13 morphological traits for both C. lasiophyllus lineages, C. flavescens, and C. anceps. In (A), principal component axis one (PC1) and PC2 explain 34.6% and 18.3% of the variation, respectively. In (B), PC2 and PC3 are compared. PC3 explains an additional 14.0% of the variation. [file AJB2-107-1815-s003.docx]

Appendix S3. Appendix S3. Principal components analysis of 13 morphological traits for both *C. lasiophyllus* lineages, *C. flavescens* and *C. anceps*. In (A), principal component axis one (PC1) and PC2 explain 34.6% and 18.3% of the variation, respectively. In (B), PC2 and PC3 are compared. PC3 explains an additional 14.0% of the variation.
