# Supplementary material for: Two cryptic species of California mustard within Caulanthus lasiophyllus
Source: Am J Bot. 2020 Dec 28;107(12):1815–30. doi: 10.1002/ajb2.1562 (PMC7839454; doi:10.1002/ajb2.1562)
Supplement: Supplementary file 4 — APPENDIX S4. Climate niche data for two types of C. lasiophyllus samples. Violin plots for the northern/coastal samples (gray fill) and southern/inland samples (no fill) are from PRISM’s 30 year normals (1981–2010). Medians are indicated with black circles. First through third quartiles are indicated with thickened black, vertical lines. Months are listed in calendar order abbreviated by their first letter followed by annual values. (A) Total precipitation by month and for the annual total (Ann). Note: separate y‐axis for annual total on right. (B) Mean temperature by month and for the annual total (Ann). [file AJB2-107-1815-s004.docx]

Appendix S4. Climate niche data for two types of *C. lasiophyllus* samples. Violin plots for the northern/coastal samples (gray fill) and southern/inland samples (no fill) are from PRISM’s 30 year normals (1981-2010). Medians are indicated with black circles. First through third quartiles are indicated with thickened black, vertical lines. Months are listed in calendar order abbreviated by their first letter followed by annual values. (A) Total precipitation by month and for the annual total (Ann) – note: separate y-axis for annual total on right. (B) Mean temperature by month and for the annual total (Ann).
